# Low *in vitro* activity of sertaconazole against clinical isolates of dermatophyte 

**DOI:** 10.18502/cmm.6.1.2507

**Published:** 2020

**Authors:** Mahsa Behnam, Hossein Zarrinfar, Mohammad Javad Najafzadeh, Ali Naseri, Lida Jarahi, Monika Novak Babič

**Affiliations:** 1 Department of Parasitology and Mycology, School of Medicine, Mashhad University of Medical Sciences, Mashhad, Iran; 2 Allergy Research Center, Mashhad University of Medical Sciences, Mashhad, Iran; 3 Department of Community Medicine, Faculty of Medicine, Mashhad University of Medical Sciences, Mashhad, Iran; 4 Department of Biology, Biotechnical Faculty, University of Ljubljana, Ljubljana, Slovenia

**Keywords:** Antifungal drugs, Dermatophytes, Mashhad, Microdilution, Sertaconazole

## Abstract

**Background and Purpose:**

Dermatophytes are a group of fungi specialized in invading humans and other vertebrate keratinized tissues. These fungi cause a variety of skin, nail, and hair disorders, called dermatophytosis (tinea). In some cases, drug resistance to antifungals necessitates special treatment. Among the antifungal agents, *sertaconazole (i.e., a *third-generation imidazole) has a broad-spectrum against dermatophyte species. Regarding this, the present study was conducted to investigate the antifungal susceptibility of dermatophytes obtained from patients with dermatophytosis in Mashhad located in northeastern Iran.

**Materials and Methods:**

A total of 75 clinical dermatophyte isolates, including *Trichophyton mentagrophytes* (n=21), *T. interdigital *(n=18), *T. tonsurans *(n=16), *Epidermophyton floccosum *(n=11), *Microsporum canis* (n=5), *Nannizzia fulvum *(n=2), *T. benhamiae *(n=1), and *T. verrucosum *(n=1), were evaluated against five antifungal agents of *sertaconazole*, itraconazole, clotrimazole, terbinafine, and griseofulvin based on the CLSI M38-A2 guideline.

**Results:**

**According to the results, the minimum inhibitory concentration (MIC) ranges of** *sertaconazole*, terbinafine, griseofulvin, itraconazole, and clotrimazole **were estimated at** 0.125-16, 0.002-1, 0.5-4, 0.031-4, and 0.016-4 µg/ml, respectively, **for dermatophyte species**. In addition, the geometric mean (GM) values of the MIC of *sertaconazole*, terbinafine, griseofulvin, itraconazole, and clotrimazole were obtained as 3.39, 1, 1.44, 1.52, and 1.93, respectively.

**Conclusion:**

Among the tested antifungals, terbinafine and griseofulvin were the most effective agents against dermatophyte isolates. However, sertaconazole, a third-generation imidazole, did not show any significant effect. Furthermore, M. canis and E. floccosum showed the best response to the antifungal agents.

## Introduction

Dermatophytes are a group of keratinophilic fungi that often invade human and vertebrate keratinized tissues. These fungi are capable of causing various disorders in the skin, nails, and hair, as well as inducing deep tissue invasion in some cases [1]. Due to the advances in the accurate identification of dermatophytes and phylogenetic changes in recent years, approximately eight genera (i.e., *Arthroderma, Epidermophyton, Guarromyces, Lophophyton, Microsporum, Nannizzia*, *Paraphyton*, and* Trichophyton*) and more than 50 species of dermatophytes have been introduced globally [2]. The global prevalence of dermatophytosis is estimated to be around 20% [3]. 

Not only do dermatophytes cause general health problems but also some of the species express various susceptibility to antifungal drugs [4]. Although the skin lesions of dermatophytosis usually respond well to the routine treatments with topical antifungal drugs, they might be often chronic and do not respond well to the usual therapeutic procedure [5]. There are reports regarding drug resistance in the treatment of tinea capitis [6], one of which was terbinafine (TER) resistance in onychomycosis caused by *T. rubrum* [7]. These cases can lead to unsuccessful or prolonged therapies and cause additional complications (e.g., increased medical costs and possible side effects of used antifungal drugs) for patients [8]. 

Causes of treatment failure frequently include peripheral vascular disease, resistant structures (*pseudomycetoma*), presence of dormant fungal spores, and failure to continue treatment by patients [8]. In recent years, studies have been conducted to determine antifungal susceptibility by testing the clinical isolates of dermatophytes in some areas. However, these studies have often been limited to specific dermatophytes and the available *antifungals *[9]. Among the antifungal agents, *sertaconazole (STZ) as the *third-generation imidazole has a broad-spectrum against dermatophyte species, *various* yeasts, filamentous fungi, and even some bacteria [10]. Nonetheless, there is no available information regarding the *in vitro *activity of STZ against dermatophytes. In some parts of the world, various studies reported different values for this agent in clinical and non-clinical domains [11, 12]. Therefore, the determination of antifungal susceptibility of dominant fungal groups obtained from clinical samples collected from a large area seems necessary. 

With this background in mind, the present study was conducted to investigate the activity of five antifungal agents, namely *STZ*, itraconazole (ITC), TER, clotrimazole (CLO), and griseofulvin (GRI), against eight species of clinical dermatophytes obtained from patients with dermatophytosis (ringworm or tinea) in Mashhad, northeastern Iran. 

## Materials and Methods

This study was approved by the Ethics Committee of Mashhad University of Medical Sciences, Mashhad, Iran, with the Ethics Committee code of IR.MUMS. fm.REC.1396.406. 

***Fungal strains***

The dermatophytic fungi were previously identified based on morphological characteristics and *DNA sequence*-*based method* (rDNA *ITS* region) [13, 14]. The dermatophyte isolates included eight species of *T. mentagrophytes* (n=21), *T. interdigital *(n=18), *T. tonsurans *(n=16), *E. floccosum *(n=11), *M. canis* (n=5), *N. fulvum *(n=2), *T. benhamiae *(n=1), and *T. verrucosum *(n=1). All isolates were obtained from patients with dermatophytosis in Mashhad city. The specimens were collected from the skin, nails, and hair.

***In vitro*** ***antifungal susceptibility testing***

*Antifungal susceptibility testing* was performed according to the CLSI M38A2 guidelines, using five antifungals, namely *STZ *(Fanavaran Daroui Hakim, FDH co.)*,* ITC (Fanavaran Daroui Hakim, FDH co.), CLO (Fanavaran Daroui Hakim, FDH co.), TER (Fanavaran Daroui Hakim, FDH co.), and GRI (Sigma). The antifungal powders were dissolved to prepare the stock solution using *dimethylsulfoxide* solvent. The stock solutions of antifungals were then prepared in a culture medium RPMI-1640 (Sigma) in 96-well microplates at the final concentrations of 0.016-16, 0.002-4, and 0.008-8 µg/ml for *STZ*/ITC/CLO, TER, and GRI, respectively. All dermatophyte isolates were cultured on potato dextrose agar (Sigma, Germany) and stored at 30°C for 1-2 weeks. Fungal suspensions were prepared using sterile swabs to harvest spores from sporulated colonies. The swabs were then transferred to a sterile saline solution with the addition of Tween 20. 

The suspensions containing harvested conidia and hyphal fragments were vortexed for 30 sec and stored at room temperature for 10 min. Fungal suspensions were evaluated via the spectrophotometer at a wavelength of 530 nm to reach a 65-70% transmittance and then were diluted 1:50 in RPMI 1640 medium to achieve the final concentrations (1-3×103 CFU/ml). In the 96-well microplates, the inocula, along with the indicated concentrations of antifungals, were incubated at 35°C for 3-5 days. Incubation was prolonged for another week in cases where the positive control expressed no growth. 

After incubation, the minimum inhibitory concentration (MIC) of each antifungal drug was established by comparing the growth of positive controls and fungi in the test wells. The **MICs were determined visually as the lowest concentration of each antifungal drug that resulted in at least 80% ***growth ***inhibition, ***compared* to **the*** growth of the control* well. **The **MIC_50_ and MIC_90_ were defined as the minimum concentration at which 50% and 90% of the isolates were inhibited, respectively. The average geometric mean (GM) of the MICs of the various drugs and differences between the mean values were determined using the SPSS software (version 16).

## Results

**Figure 1:**
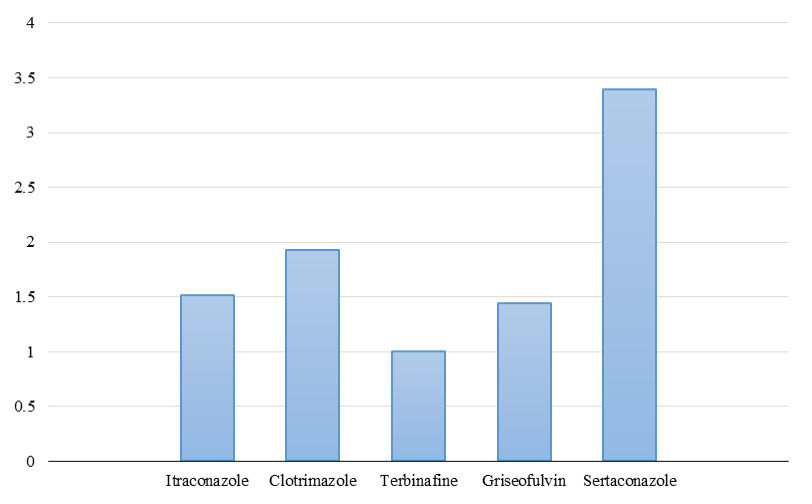
Geometric mean of five antifungals of itraconazole (1.52), clotrimazole (1.93), terbinafine (1), griseofulvin (1.44), and sertaconazole (3.39) against dermatophyte isolates in Mashhad, northeast Iran

According to the results, the lowest MIC range was observed for TER with a concentration of 0.002-1 μg/ml, followed by CLO, ITC, GRI, and *STZ* with the concentrations of 0.016-4, 0.031-4, 0.5-4, and 0.125-16 µg/ ml, respectively. Accordingly, TER had also the lowest MIC_50_ observed at a concentration of 0.125 μg/ml. The MIC_50_ determined for CLO, ITC, GRI, and *STZ* were at the concentrations of 0.5, 1, 1, and 4 μg/ml, respectively. The TER was observed to have also the lowest MIC_90_ with a concentration of 1 μg/ml, followed by CLO, ITC, GRI, and *STZ* with the concentrations of 2, 2, 2, and 8 μg/ml, respectively. The detailed information on the *results* of *antifungal susceptibility* testing against the dermatophyte isolates is shown in Table 1. The antifungal GM of TER, GRI, ITC, CLO, and *STZ* were estimated at 1, 1.44, 1.52, 1.93, and 3.39, respectively (Figure 1).

Generally, among the tested antifungals, TER and GRI were the most effective agents against dermatophyte isolates. Moreover, our results indicated no significant differences between dermatophyte isolates regarding susceptibility to the antifungals. However, M. canis and E. floccosum showed the best response to the antifungal agents.

**Table 1.** Species distribution and antifungal susceptibility profiles among dermatophyte isolates in Mashhad, northeast Iran

**MIC**_90_ **(µg/mL)**	**MIC**_50_ **(µg/mL)**	**MIC range (µg/mL)**	**Antifungal drug**	**No. (%)**	**Dermatophyte species**
2	1	0.25-2	Itraconazole	21 (28%)	Trichophyton* mentagrophytes*
2	0.5	0.063-4	Clotrimazole		
0.5	0.5	0.002-1	Terbinafine		
4	1	0.5-4	Griseofulvin		
16	4	0.5-16	sertaconazole		
2	1	0.063-2	Itraconazole	18 (24%)	*T. interdigital*
2	0.5	0.016-4	Clotrimazole		
0.5	0. 125	0.002-1	Terbinafine		
2	2	0.5-4	Griseofulvin		
8	4	0.125-16	sertaconazole		
2	0.5	0.031-2	Itraconazole	16 (21.3%)	*T. tonsurans*
2	0. 25	0.031-4	Clotrimazole		
0.5	0. 125	0.002-0.5	Terbinafine		
2	1	0.5-2	Griseofulvin		
8	2	0.25-8	sertaconazole		
2	2	0.25-2	Itraconazole	11 (14.7%)	*Epidermophyton floccosum*
1	0.5	0.125-2	clotrimazole		
0.5	0.125	0.125-1	Terbinafine		
1	1	0.5-1	Griseofulvin		
4	2	1-16	sertaconazole		
1	1	0.031-1	Itraconazole	5 (6.7%)	*Microsporum canis*
0.125	0.125	0.063-0.25	clotrimazole		
0.125	0.063	0.002-0.125	Terbinafine		
1	0.5	0.5-2	Griseofulvin		
2	2	1-4	sertaconazole		
-	-	0.5	Itraconazole	2 (2.7%)	*Nannizia* * fulvum*
-	-	0.25-0.5	clotrimazole		
-	-	1	Terbinafine		
-	-	2	Griseofulvin		
-	-	1-2	sertaconazole		
-	-	2	Itraconazole	1 (1.3%)	*T. benhamiae*
-	-	2	Clotrimazole		
-	-	1	Terbinafine		
-	-	1	Griseofulvin		
-	-	8	Sertaconazole		
-	-	4	IItraconazole	1 (1.3%)	*T. verrocosum*
-	-	4	Clotrimazole		
-	-	1	Terbinafine		
-	-	0.5	Griseofulvin		
-	-	8	sertaconazole		
2	1	0.031-4	Itraconazole	75 (100%)	Dermatophyte isolates
2	0.5	0.016-4	Clotrimazole		
1	0.125	0.002-1	Terbinafine		
2	1	0.5-4	Griseofulvin		
8	4	0.125-16	Sertaconazole		

MIC: minimum inhibitory concentration

## Discussion

The high prevalence of dermatophytosis in some areas, as well as the prolonged treatment for some clinical forms of dermatophytosis, imposes elevated treatment costs [15]. The present study was targeted toward evaluating the activity of five antifungal agents, namely STZ, ITC, TER, ClO, and GRI, against the clinical isolates of dermatophytes obtained from patients affected by dermatophytosis in Mashhad. In general, all antifungal agents exhibited good activity against all tested isolates (MIC50=0.125-4 μg/ml). However, TER and STZ showed higher and lower potency with the MIC50 values of 0.125 and 4 μg/ml, respectively.

Accurate identification of dermatophyte agents before initiating the treatment of dermatophytosis can be very effective in reducing the duration of treatment, thereby decreasing the adverse drug effects in patients and mitigating the problem of drug resistance. Furthermore, the detection of species and identification of anthropophilic or zoophilic isolates may be necessary for epidemiological studies and prevention planning concerning the source of infection [15]. Although some studies have suggested the use of specific antifungals for specific dermatophyte species, the same antifungal agent can be used in the treatment of other dermatophytes [16]. There is little information about the use of STZ for dermatophytosis, especially in Iran. However, some new antifungals, such as luliconazole and lanoconazole, showed high activity against dermatophyte species [17, 18]. 

Nevertheless, several studies have shown that the different types of dermatophytes have different sensitivities to antifungal drugs [19, 20]. Even the species causing the infection might respond differently during the therapeutic process than during the *in vitro* susceptibility testing of antifungals. The location of the lesions can influence the factors accounting for the observed differences [21]. Although some types of mild to moderate dermatophytosis can be managed with topical antifungal agents, particular cases, such as tinea unguium and tinea capitis, require systemic treatment. Therefore, the determination of the antifungal susceptibility pattern of dermatophytes is crucial for the physicians to prescribe the *right dose of the selected drug* in order to treat patients with a different range of diseases. 

Moreover, the determination of antifungal susceptibility *facilitates the evaluation of the potential of new drugs in the treatment of dermatophytosis [*19*].* *Based on the results of the present study, all dermatophyte isolates were more susceptible to TER than to the used azoles. This is in accordance with the results of the previous studies conducted by Silva et al. (2013) in Brazil [*22*] and Nathan et al. (2014) in the United States [*23*], reporting TER to be more effective than other antifungal drugs.* However*, Mukherjee et al. (2003) reported a case of T. rubrum that was resistant to TER with the MIC of > 4 μg/ml. This may occur due to the resistance of the tested strain that could not be confirmed by our results given its unavailability to be examined in the present study.* *In a study carried out by Gannoum et al. (2006), comparing the topical effects of TER with those of other drugs, TER was again reported to have a better antifungal effect [*24*].* *Although the method of the present study differs from that adopted by Gannoum et al. (2006), the same results were obtained in the current research, indicating TER as the best anti-dermatophyte drug available at the moment.*

In a study, *Ansari et al. (2016) investigated the antifungal effects of TER, ITC, GRI, and fluconazole against dermatophytes using the CLSI-M38-A2 protocol [*25*]. They introduced TER as the most effective drug with the lowest MIC, followed by ITC and GRI, which is almost entirely consistent with the adopted protocol, antifungals, and results obtained in our study.* *In a study performed by Badali et al. (2015) in Mazandaran, Iran, investigating the effects of antifungal drugs based on the M38-A2 protocol, TER was reported to be more efficient than azole and echinocandins and recognized as the best antifungal agent [*26*].* *It appears that different species have shown good susceptibility to TER, indicating the lack of resistance in tested species and isolates obtained from different geographical areas.*

*However, in a study carried out by Afshari et al. (2016) in Tehran, Iran [* 27 *], TER was reported to be the most effective medication against dermatophyte isolates after *ITC*, which is slightly contradictory to the results of the present study.* *The reported MIC*_50_* of TER in the *T. interdigital *(0.5 μg/ ml) was consistent with the MIC*_90_* obtained in our study.* *Similarly, the MIC*_50_* of TER reported for *E. floccosum (*0.5 μg/ml) was consistent with our MIC*_90_*. In addition, in a study conducted by Adimi et al. (2013) in Tehran [*28*], ITC was recognized as the most effective antifungal against dermatophyte isolates. *

*These differences between the observed susceptibility in the studied isolates may be due to different geographical locations and climatic conditions.* In this regard, environmental conditions can affect the expression of certain genes and production of specific enzymes in fungi [29]. *Furthermore, the number of isolates and diversity of the studied species can* fundamentally *influence* the *results*. In the mentioned study, fluconazole yielded the highest MIC and GM among the dermatophyte isolates, which may indicate increased resistance to this drug in recent years. Therefore, the use of this medication in the treatment of dermatophytosis should be evaluated with caution [28]. Although in the present study, *TER and ITC showed an effective activity against dermatophyte isolates, their *GM MICs were so high that this difference remained largely unknown. It might be due to the differences between strains or phenotype and genotype diversity, number of fungi tested, and drug potency. However, these are only some hypotheses, and more work needs to be done to prove them. 

During the past years in some areas, *GRI* has been used as the first antifungal line against dermatophyte infections. In our study, outstanding results were obtained with regard to MIC_90_ (2 μg/ml) and GM (1.44 μg/ml), respectively. However, the previous studies performed by Chadeganipour *et al*. *(2004)* [20] and Adimi *et al*.* (2013)* [28] reported an MIC_90_ **range of** 8-256 μg/ml, indicating possible drug resistance in the studied isolates. The reason for the observed discrepancy may be related to the genotypic differences occurring in some species and expression of drug-resistance genes [30]. 

In a study, Falahati et al. (2018) [31] investigated the effects of two drugs, namely GRI and TER, on eight dermatophyte species. Their results indicated that TER was more efficient against the tested dermatophytes, which is consistent with the results obtained by Ghannum et al. (2006) [32] and our results where TER had a higher GM than GRI. In another study carried out by Baghi et al. (2016) [17], GRI showed low susceptibility, compared to the new antifungals, such as luliconazole and lanoconazole. Among the dermatophyte isolates examined in the present study, the widest MIC range of GRI was observed for T. mentagrophytes (0.5-4 μg/ml) that is higher than those obtained for TER, ITC, and CLO. However, the MIC50 (1 μg/ml) of GRI and ITC was equal to and higher than those of TER and CLO, respectively. 

The STZ, a third-generation imidazole, has a comprehensive action spectrum on various fungi [10]. However, in the current study, this antifungal had a weaker effect than other antifungals against dermatophyte isolates with a GM of 3.39. On the other hand, there is limited information on the effect of this medication on dermatophyte isolates in Iran. Although in some reports, this drug was more effective against the resistant strains of dermatophytes than other antifungals, these results are inconsistent with our findings [10, 33]. Moreover, in this study, the widest MIC range of STZ was observed for T. interdigital species (0.125-16 μg/ml) among the dermatophyte isolates.

However, this drug had a significant effect on other yeasts and filamentous fungi [10]. In a study carried out by Shivamurithy et al. (2014) in India [34], the effect of CLO and STZ on tinea corporis was investigated, and STZ showed a better effect during the treatment process than CLO. This result was inconsistent with those obtained in the present study, which may be due to the differences in the method of antifungal sensitivity testing. Ferrnandez et al. (2003) [35] used a similar microdilution broth method as was used in our study. Their results for CLO showed an MIC50 for T. tonsurans at a concentration of 0.25 µg/ml, which is entirely consistent with our study. In the present study and among the dermatophyte isolates, the narrowest MIC range of CLO was obtained for M. canis (0.063-0.25 μg/ml). Moreover, CLO showed an MIC50 (0.5 μg/ml) lower than those of ITC, GRI, and STZ. 

The present study was the first attempt investigating the antifungal susceptibility profile of dermatophyte isolates obtained from dermatophytosis in Mashhad. However, it also contains some limitations, including the low number of clinical isolates of dermatophytes and restricted use of antifungal drugs. Therefore, it is required to perform further studies using a higher number of clinical isolates and more antifungal medications. However, the comprehensive consideration of these small, cross-sectional studies can provide some useful information on the susceptibility pattern of different drugs to dermatophyte isolates in the different regions of Iran. On the other hand, more widespread information can be obtained by following up the treatment of these patients and comparing them with laboratory results.

## Conclusion

As the results of the present study indicated, TER and GRI were respectively the most effective antifungals against dermatophyte isolates among the tested antifungal drugs. The lowest yield was related to *STZ*; however, it also had a productive activity against dermatophyte isolates. Furthermore, none of the dermatophyte isolates showed high MIC (resistance) to these drugs. Additionally, *M. canis* and *E. floccosum* showed the lowest GM (the highest drug sensitivity) to antifungal medications.
